# Monoubiquitination in Homeostasis and Cancer

**DOI:** 10.3390/ijms23115925

**Published:** 2022-05-25

**Authors:** Yujie Chen, Dandan Zhou, Yinan Yao, Yutong Sun, Fan Yao, Li Ma

**Affiliations:** 1 Hubei Hongshan Laboratory, College of Biomedicine and Health, Huazhong Agricultural University, Wuhan 430070, China; yjchen@webmail.hzau.edu.cn (Y.C.); zoeych@webmail.hzau.edu.cn (D.Z.); yinanyao@webmail.hzau.edu.cn (Y.Y.); 2 Department of Molecular and Cellular Oncology, The University of Texas MD Anderson Cancer Center, Houston, TX 77030, USA; ysun2@mdanderson.org; 3 Hubei Clinical Research Center for Precise Diagnosis and Treatment of Liver Cancer, Taihe Hospital, Hubei University of Medicine, Shiyan 442000, China; 4 Department of Experimental Radiation Oncology, The University of Texas MD Anderson Cancer Center, Houston, TX 77030, USA; 5 The University of Texas MD Anderson UTHealth Graduate School of Biomedical Sciences, Houston, TX 77030, USA

**Keywords:** monoubiquitination, homeostasis, cancer

## Abstract

Monoubiquitination is a post-translational modification (PTM), through which a single ubiquitin molecule is covalently conjugated to a lysine residue of the target protein. Monoubiquitination regulates the activity, subcellular localization, protein–protein interactions, or endocytosis of the substrate. In doing so, monoubiquitination is implicated in diverse cellular processes, including gene transcription, endocytosis, signal transduction, cell death, and DNA damage repair, which in turn regulate cell-cycle progression, survival, proliferation, and stress response. In this review, we summarize the functions of monoubiquitination and discuss how this PTM modulates homeostasis and cancer.

## 1. Introduction

Ubiquitination is one of the most important post-translational modifications (PTMs) of proteins in maintaining cellular and physiological homeostasis [1]. This dynamic process is characterized by the sequential transfer of ubiquitin, a ubiquitously expressed protein consisting of 76 amino acids, to target proteins by three enzymes: E1, ubiquitin-activating enzyme; E2, ubiquitin-conjugating enzyme; E3, ubiquitin ligase. The reverse process, deubiquitination, or the removal of ubiquitin from target proteins, is catalyzed by deubiquitinating enzymes (DUBs). In humans, only two E1 ubiquitin-activating enzymes are found, whereas approximately 40 E2s, 600 E3s, and 100 DUBs have been identified [2,3,4].

On the basis of their constituent domains and ubiquitin transfer mechanisms, ubiquitin ligases are classified into three families: RING (really interesting new gene), HECT (homologous to the E6AP carboxyl terminus), and RBR (RING-between-RING) [2,5]. RING E3s, the largest family of ubiquitin ligases, act as scaffolds to directly transfer ubiquitin from E2 to the target protein. The ubiquitin transfer event mediated by HECT E3s begins with transthiolation, moving the ubiquitin molecule from the E2 to the E3. The ubiquitin transfer mechanism of RBR E3s is a hybrid of RING and HECT E3s, where the RING1 domain recruits a ubiquitin-charged E2 and transfers ubiquitin to the catalytic cysteine residue in the RING2 domain before its conjugation to a protein substrate [2]. The linkage specificity varies among these three E3 families. Whereas the ubiquitin linkage types of HECT and RBR E3s are determined by themselves, RING E3s rely on the E2 to deliver the linkage specificity [2].

DUBs are classified into seven families [6]: ubiquitin-specific proteases (USPs), ubiquitin C-terminal hydrolases (UCHs), ovarian tumor proteases (OTUs), Machado–Joseph disease proteases (MJDs), JAB1/MPN/MOV34 metalloenzymes (JAMMs), motif interacting with ubiquitin-containing novel DUB family (MINDYs), and zinc finger with UFM1-specific peptidase domain proteins (ZUFSPs). DUBs can also be categorized into cysteine proteases and zinc metalloproteases. USPs, UCHs, OTUs, MJDs, MINDYs, and ZUFSPs belong to the former type, whereas JAMMs belong to the latter [6]. DUBs can cleave ubiquitin chains internally (*endo*) or from the end (*exo*). The proteasome-associated deubiquitinase USP14 has *exo*-activity, as it cleaves K48-linked polyubiquitin chains from the distal end, giving rise to monoubiquitin [7]. On the contrary, DUBs that regulate ubiquitin-mediated signaling, such as CYLD, A20, and USP9X, have *endo*-activity, liberating polyubiquitin chains from the substrates via internal cleavage [8,9,10].

**Figure 1:**
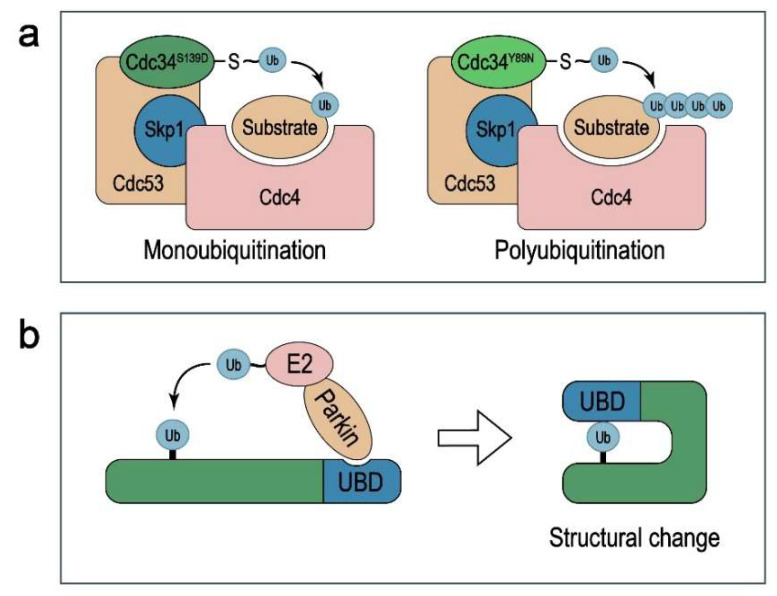
The models for mechanisms that dictate whether a protein is monoubiquitinated or polyubiquitinated. (**a**) The amino acids in the catalytic region of E2s and their compatibility with the residues surrounding the lysine residues in the substrate and ubiquitin serve as the determinants. For example, key residues in the catalytic core of the yeast E2–E3 complex Cdc34–SCF^Cdc4^ dictate the lysine preference of Cdc34 and whether this complex monoubiquitinates or polyubiquitinates its substrate. (**b**) The structural changes of the monoubiquitinated substrate restrict its further polyubiquitination. For example, the ubiquitin-binding domain (UBD) of the adaptor protein Eps15 is blocked after being monoubiquitinated by the RING-family E3 ligase Parkin, which limits ubiquitin chain extension.

Proteins can be ubiquitinated in three ways, including monoubiquitination, multi-ubiquitination, and polyubiquitination, and different modifications exert different functions [11,12]. Whereas Lys48 (K48)-linked polyubiquitination typically results in proteasomal degradation of the target protein, monoubiquitination has been reported to confer stability to the substrate by inhibiting its polyubiquitination and degradation [13]. For instance, the HECT-type E3 ligase NEDD4-1 can promote both mono- and polyubiquitination of PTEN, with monoubiquitination protecting PTEN through nuclear import and polyubiquitination leading to its degradation in the cytoplasm [14]. The best-studied non-proteolytic ubiquitination is Lys63 (K63)-linked polyubiquitination, which regulates the substrate’s enzymatic activity (e.g., kinase activity) [15] or subcellular localization [16]. The mechanisms that dictate whether a protein is monoubiquitinated or polyubiquitinated are not completely understood. One possibility is that the amino acids in the catalytic region of E2s and their compatibility with the residues surrounding the lysine residues in the substrate and ubiquitin serve as the determinants. In favor of this possibility, key residues in the catalytic core of the yeast E2–E3 complex Cdc34–SCF^Cdc4^ dictate the lysine preference of Cdc34 and whether this complex monoubiquitinates or polyubiquitinates its substrate [17,18] (Figure 1a). Another possible mechanism is that the structural changes of the monoubiquitinated substrate restrict its further polyubiquitination. For example, the ubiquitin-binding domain (UBD) of the adaptor protein Eps15 is blocked after being monoubiquitinated by the RING-family E3 ligase Parkin, which limits ubiquitin chain extension [19] (Figure 1b).

Monoubiquitin conjugation on a single residue or multiple sites can alter the subcellular localization [20], protein–protein interactions [21], endocytosis [22], and activity [23,24] of the substrate. In doing so, monoubiquitination is implicated in many cellular processes, such as gene transcription [25,26,27], signal transduction [28], and DNA damage response [29,30,31]. At the molecular level, monoubiquitinated proteins bind proteins containing UBDs. For instance, monoubiquitination of proliferating cell nuclear antigen (PCNA) serves as a binding platform for the Y-family translesion synthesis (TLS) polymerases, which contain two types of C-terminal UBDs, ubiquitin-binding zinc fingers (UBZs) and ubiquitin-binding motifs (UBMs), allowing cells to replicate across DNA lesions [32]. Notably, many proteins that contain UBDs (referred to as ubiquitin receptors), such as the ubiquitin-interacting motif (UIM), are often themselves monoubiquitinated, referred to as coupled monoubiquitination [33].

Thus far, protein monoubiquitination is much less studied compared with polyubiquitination. Interestingly, a quantitative proteomic analysis using isotope-labeled ubiquitin revealed that, among ubiquitinated proteins in cells, more ubiquitin molecules are present as monoubiquitinated proteins compared with polyubiquitinated ones [34]. In this review, we summarize the current knowledge about the roles of monoubiquitination in cellular functions and homeostasis, and we discuss how its disruption contributes to diseases including anemia, neurodegeneration, and cancer.

## 2. Protein Substrates of Monoubiquitination

### 2.1. Histones H2A and H2B

**Figure 2:**
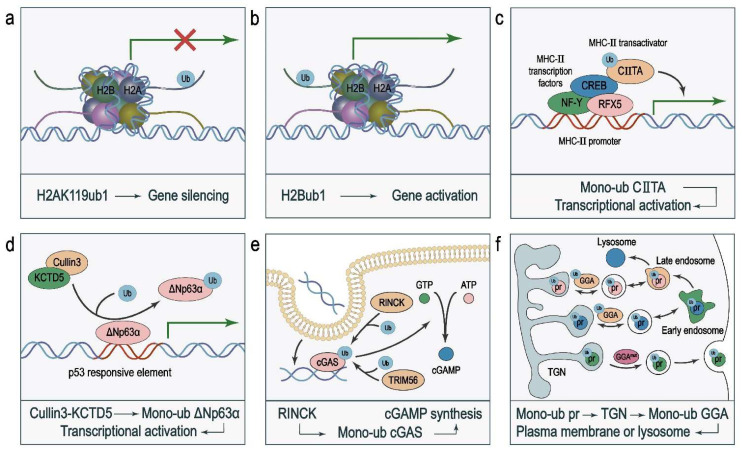
Protein substrates of monoubiquitination. (**a**) H2AK119ub1 mediates gene silencing. (**b**) H2Bub1 mediates transcriptional activation. (**c**) The MHC-II transactivator CIITA is recruited by MHC-II transcription factors (RFX5, CREB, and NF-Y) to the promoters to activate the transcription of MHC-II genes. Monoubiquitination of CIITA enhances its interaction with both transcription factors and the MHC-II gene promoters, leading to transcriptional activation. (**d**) ΔNp63α competitively binds to p53-responsive elements to repress the expression of p53 target genes. Cullin3–KCTD5 monoubiquitinates ΔNp63α to decrease its DNA-binding affinity, leading to reduced transcriptional repressor activity. (**e**) RINCK or TRIM56 positively regulates cGAMP synthesis by mediating the monoubiquitination and activation of cGAS. (**f**) Monoubiquitination marks proteins (pr) for sorting via the trans-Golgi network (TGN). The Golgi-localized, γ-ear-containing, Arf (ADP-ribosylation factor)-binding (GGA) proteins are clathrin adaptors that bind ubiquitin via their GAT domain to control the trafficking between the TGN, endosome, and plasma membrane or lysosome.

Histone octamers, composed of H2A–H2B and H3–H4 dimers, are protein products of the most conserved gene family in eukaryotes [35]. PTMs of histones, including acetylation [36], methylation [37], phosphorylation [38], and ubiquitination [39,40], can affect chromatin structure, transcription, and genome stability. Monoubiquitination of histones plays an important role in epigenetic regulation of gene transcription [25,26,27,41]. The most common histone monoubiquitination occurs on Lys119 of histone H2A (H2AK119ub1) [42], which is mainly catalyzed by its writer polycomb repressive complex 1 (PRC1) and removed by its eraser complex composed of ASX-like (ASXL) and BAP1 [43,44,45]. H2AK119ub1 is associated with gene silencing (Figure 2a). It has been shown that ASXL1 binds and deubiquitinates H2AK119ub1 at the promoter of *PTEN* for its transcriptional activation, and that loss-of-function mutation or downregulation of ASXL1 leads to downregulation of PTEN, activation of AKT, and increased cell proliferation [46].

On the contrary, another monoubiquitinated histone protein, H2Bub1, is associated with increased transcriptional activity [47,48] (Figure 2b), and loss of H2B monoubiquitination correlates with malignant progression in lung cancer [49]. Mechanistically, H2Bub1 not only recruits transcription factors but also opens chromatin structure and makes it accessible to transcription factors and other protein complexes. Loss of H2Bub1 leads to downregulated expression of mitochondrial respiratory genes, which in turn inhibits the oxidative phosphorylation pathway [50]. Consequently, cancer cells need to generate more energy to support their survival [51]. This finding reveals a mechanism via which histone monoubiquitination regulates the Warburg effect and energy metabolism of cancer cells.

### 2.2. Transcription Factors and Other Transcriptional Regulators

Monoubiquitination of transcription factors can modulate gene transcription by regulating protein–protein interaction, protein–DNA binding, and nuclear translocation. It has been shown that ubiquitin-mediated binding of transcriptional activators to gene promoters activates gene transcription. For instance, the major histocompatibility complex (MHC) class II transactivator, CIITA, which is recruited by MHC-II transcription factors (RFX5, CREB, and NF-Y) to the promoters of the three MHC-II genes, is monoubiquitinated. This ubiquitination of CIITA enhances its binding to both MHC-II transcription factors and the MHC-II gene promoters, leading to their transcriptional activation [21] (Figure 2c).

Monoubiquitination regulates protein–DNA interactions. ΔNp63α, a member of the p53 family, competitively binds to p53-responsive elements to repress the expression of p53 target genes, such as p21, leading to increased cell survival and proliferation [52]. The ubiquitin ligase complex Cullin3–KCTD5 monoubiquitinates ΔNp63α to decrease its DNA-binding affinity, leading to reduced transcriptional repressor activity of ΔNp63α [53] (Figure 2d). KCTD5 is a component of a Cullin–RING E3 ubiquitin ligase (CRL) complex. Structural studies revealed that the substrate-binding component of the CRL complex can orient its substrates for ubiquitination [54].

The nuclear translocation of transcription factors, which can be regulated by monoubiquitination, is important for their transcriptional activity. For instance, oxidative stress in cells induces monoubiquitination of Forkhead box O (FOXO) transcription factors, leading to their nuclear import and enhanced transcriptional activity [55].

### 2.3. Enzymes

The catalytic activity of enzymes is modulated by PTMs including monoubiquitination. For instance, the paracaspase MALT1 activates NF-κB signaling through its protease activity [56]. In activated T lymphocytes, MALT1 is monoubiquitinated at Lys644 in the C terminus, leading to induction of its protease activity [24]. Lys644 is located in an exposed loop structure of the immunoglobulin fold beyond the caspase-like domain, and the crystal structure suggests that monoubiquitination at Lys644 can induce homodimerization of the caspase-like domain, which in turn activates the enzyme [57]. Another example is the histone H3K9 methyltransferase SETDB1. SETDB1 is constitutively monoubiquitinated at Lys867, and K867ub1 is instrumental in its catalytic activity [58]. Cyclic guanosine monophosphate–adenosine monophosphate synthase (cGAS), a cytosolic DNA sensor in the cGAS–STING signaling pathway, recognizes double-stranded DNA in the cytoplasm, resulting in elevated expression of type I interferons (IFNs) and other immune regulatory molecules via an enzyme cascade reaction. Two E3 ubiquitin ligases, TRIM56 and TRIM41 (RINCK), monoubiquitinate cGAS to activate cGAS, stimulate cGAMP synthesis, and boost antiviral innate immunity [59,60] (Figure 2e). A possible mechanism of cGAS activation by monoubiquitination is that cGAS monoubiquitination may stabilize the ternary complex formed by cGAS dimers with two DNA molecules [61]. As one of the most frequently mutated oncoproteins in human cancers, Ras acts as a molecular switch that is activated upon GTP loading and deactivated upon hydrolysis of GTP to GDP. Mutations in Ras often inhibit its GTPase activity [62]. Recent work has revealed that monoubiquitination of Ras at Lys147 promotes the release of GDP and activation of K-Ras [63].

### 2.4. Membrane Proteins

In mammalian cells, most short-lived cytosolic proteins are turned over through degradation by the proteasome [64]. However, the degradation of cell membrane proteins does not necessarily depend on polyubiquitination and proteasomal degradation; instead, monoubiquitination is often involved in this process [65]. Monoubiquitinated membrane proteins are sent to the endocytic compartments, and then directed to the lysosome for degradation or recycled to the cell surface [65]. Mechanistically, monoubiquitination marks proteins for sorting via the trans-Golgi network (TGN) [66]. Golgi-localized, γ-ear-containing, Arf (ADP-ribosylation factor)-binding (GGA) proteins, a family of clathrin adaptors, bind ubiquitin via their GAT domain to control the trafficking between the TGN, endosome, and plasma membrane or lysosome [67,68] (Figure 2f). An example is that, in yeast, the amino-acid transporter Gap1 is monoubiquitinated after internalization, and then the GGA proteins bind monoubiquitinated Gap1 to label Gap1 for sorting from the TGN to endosomes. GGA mutants that have lost the ubiquitin-binding activity fail to do so, resulting in the trafficking of Gap1 back to the cell surface [69].

## 3. Cellular Functions of Monoubiquitination

### 3.1. Transcription

Precise and dynamic regulation of transcription is essential for biological homeostasis. Chromatin remodeling processes contribute to the establishment of transcriptional states, and one of the chromatin remodeling events is histone monoubiquitination. As discussed above, H2AK119ub1 is associated with transcriptional repression [42], and H2Bub1 is associated with transcriptional activation [47,48] (Figure 2a,b).

Monoubiquitination of a transcription factor can activate transcription without targeting it for degradation. Androgen receptor (AR), a ligand-dependent transcription factor, is inactive in the cytoplasm in the absence of androgen; upon activation by its ligand, AR translocates into the nucleus and binds the promoter regions of target genes to activate their transcription [70]. Interestingly, TSG101, a co-regulator of nuclear hormone receptors, boosts AR monoubiquitination in a ligand-dependent manner, which in turn enhances AR-induced gene transcription without affecting the protein stability of AR [71].

**Figure 3:**
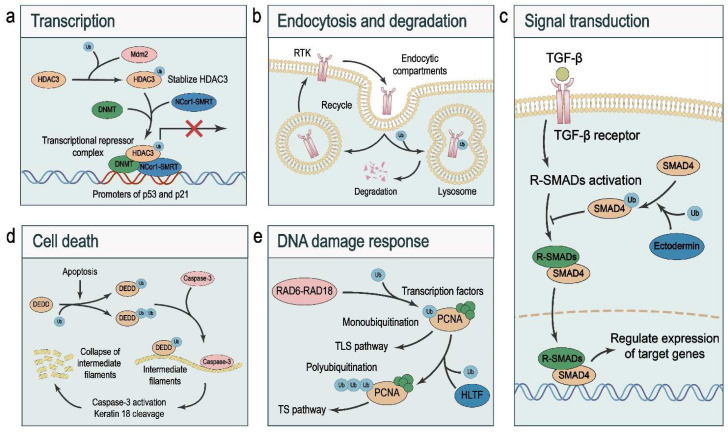
Cellular functions of monoubiquitination. (**a**) Mdm2 binds, monoubiquitinates, and stabilizes HDAC3, which forms a complex with co-repressors including DNMT and NCor1–SMRT to repress the transcription of tumor suppressors p53 and p21. (**b**) Monoubiquitinated RTKs are sorted to the lysosomal compartment, limiting their recycling to the plasma membrane. (**c**) TGF-β binds to its receptors to activate R-SMADs, which form a complex with SMAD4. The SMAD complex translocates into the nucleus to regulate target gene transcription. Monoubiquitination of SMAD4 blocks its interaction with phospho-SMAD2, resulting in inactivation of TGF-β signaling. (**d**) In response to proapoptotic stimuli, mono- and di-ubiquitinated DEDD and caspase-3 aggregate on intermediate filaments, leading to activation of caspase-3, cleavage of keratin 18, and collapse of intermediate filaments. (**e**) HLTF mediates PCNA polyubiquitination, and RAD6–RAD18 mediates PCNA monoubiquitination. In the presence of HLTF, PCNA monoubiquitination leads to its polyubiquitination, which favors the template switching (TS) pathway; in the absence of HLTF, monoubiquitinated PCNA triggers the translesion synthesis (TLS) pathway.

Monoubiquitination can also facilitate transcriptional repressors. HDAC3, one of the major epigenetic regulators, forms a complex with co-repressors including DNMT and NCor1–SMRT to repress the transcription of tumor suppressors p53 and p21. Interestingly, Mdm2 binds, monoubiquitinates, and stabilizes HDAC3, which in turn enhances HDAC-mediated transcriptional repression [72] (Figure 3a).

### 3.2. Endocytosis and Degradation

Endocytosis plays a pivotal role in maintaining the homeostasis of cell membrane compositions and mediating rapid responses to extracellular stimuli [73]. Endocytosed proteins, such as receptor tyrosine kinases (RTKs), are either recycled to the plasma membrane or directed for lysosomal degradation, resulting in distinct signaling outputs. As the last step in the negative regulation of RTKs, lysosomal degradation prevents the hyperactivation of RTKs and disruption of homeostasis, which is involved in embryonic development and the pathogenesis of diseases such as cancer [74]. RTK ubiquitination governs receptor internalization and lysosomal degradation [75]. Of note, monoubiquitination is implicated in endocytic trafficking, both during internalization and in the endosomal compartment where ubiquitinated RTKs are sorted to the lysosomal compartment, limiting their recycling to the plasma membrane [22] (Figure 3b).

In addition to lysosomal degradation, monoubiquitination can also serve as a harbinger for proteasomal degradation of target proteins. The transcription factor paired box 3 (Pax3) regulates muscle development, and its gain-of-function mutation, Pax3FKHR, causes aberrant expression of its target genes and is associated with a severe form of alveolar rhabdomyosarcoma [76]. Monoubiquitination at Lys437 or Lys475 of Pax3 promotes its proteasomal degradation [77], but no polyubiquitination of Pax3 is detected under in vivo or in vitro conditions. It is commonly thought that proteasomal degradation of proteins requires the conjugation of polyubiquitin chains [78]. The possible reason for this phenomenon is that low levels of polyubiquitinated forms of Pax3 are rapidly degraded. Alternatively, however, a recent proteomic study using yeast and human cells in which wildtype ubiquitin was replaced with ubiquitin that cannot form chains revealed that monoubiquitination can lead to proteasomal degradation, and that these monoubiquitinated substrates share unique features, such as being smaller and structurally less disordered than polyubiquitinated substrates [79].

The proteasome recognizes its ubiquitinated substrates through ubiquitin receptors including Rpn10/S5a and Rpn13 [80]. The ubiquitin receptors themselves can be modified by monoubiquitin, which modulates the recruitment of substrates to the proteasome. Rpn10 is monoubiquitinated by Rsp5 and Ubp2 at Lys71, Lys84, and Lys99 located in the VWA domain, and at Lys268 located in the C terminus of the protein, which inhibits its ability to interact with substrates, thus reducing proteasomal degradation of the substrates [23].

### 3.3. Signal Transduction

Precise control of the activity of cell signaling pathways is crucial for cellular and organismal homeostasis, and monoubiquitination has been found to regulate key signaling components in multiple pathways. Of note, the TGF-β pathway controls developmental processes, and its dysregulation is associated with the generation of cancer stem cells, extracellular matrix remodeling, tumor progression, and metastasis [81]. TGF-β binds to its receptors to activate receptor-regulated SMADs (R-SMADs), which form a complex with SMAD4. Subsequently, the SMAD complex translocates into the nucleus to regulate the transcription of its target genes [82,83]. The nuclear–cytoplasmic shuttling of SMAD4 is controlled by its PTMs, including phosphorylation and monoubiquitination [84]. The ubiquitin ligase Ectodermin/TIF1γ promotes, while the deubiquitinases USP9X and USP4 antagonize SMAD4 monoubiquitination [28,85,86]. SMAD4 is monoubiquitinated at Lys519, which inhibits SMAD4 by blocking its interaction with phospho-SMAD2, resulting in the termination of TGF-β signaling [84,85,86] (Figure 3c). I-SMADs, which consist of SMAD6 and SMAD7, are negative regulators of TGF-β and BMP signaling pathways. I-SMADs inhibit R-SMAD phosphorylation by competing with R-SMADs for receptor binding [87]. UBE2O, a ubiquitin-conjugating enzyme (E2), mediates the monoubiquitination of SMAD6 at Lys174. Compared with non-modified SMAD6, monoubiquitinated SMAD6 exhibits weaker inhibitory activity on TGF-β/BMP signaling [88].

The PI3K–AKT–mTOR signaling pathway promotes cell growth by integrating multiple extracellular signals, and its dysregulation is implicated in metabolic disorders, aging, and cancer [89]. PTEN is a lipid phosphatase that mediates the conversion of phosphatidylinositol 3,4,5-trisphosphate to phosphatidylinositol-4,5-bisphosphate, which in turn prevents hyperactivation of mTOR signaling and maintains the homeostasis in protein synthesis and cell growth [90]. PTEN can be mono- and polyubiquitinated at residues Lys13 and Lys289 by the E3 ligase NEDD4. The increase in monoubiquitination levels stabilizes PTEN by inhibiting its polyubiquitination and redistributing it to the nucleus [13].

As a central mediator of inflammatory responses, NF-κB regulates diverse aspects of immune functions, and its dysregulation is associated with inflammatory disorders and cancer [91]. SIRT6, a member of the sirtuin family, dually regulates NF-κB signaling by deacetylation and monoubiquitination. First, SIRT6 deacetylates H3K9 at the promoters of NF-κB target genes, leading to gene silencing [92]. Second, SIRT6 recruits the ubiquitin ligase SKP2 to promote monoubiquitination of the H3K9me3-specific histone methyltransferase, SUV39H1. Monoubiquitinated SUV39H1 is released from the IκBα promoter, leading to upregulation of IκBα and inhibition of NF-κB signaling [93]. The linear ubiquitin chain assembly complex (LUBAC), which is composed of HOIP, SHARPIN, and HOIL-1L, promotes the activity of NF-κB signaling. A recent study revealed that the RBR E3 ligase HOIL-1L conjugates monoubiquitin to all LUBAC subunits, leading to functional inactivation of LUBAC [94]. The introduction of E3 ligase-defective mutants of HOIL-1L into cells protected the cells from *Salmonella* infection, suggesting that inhibition of HOIL-1L is a potential strategy for treating severe infections [94].

### 3.4. Cell Death

From embryonic morphogenesis to adult tissue homeostasis, cell elimination via apoptosis or programmed cell death is an evolutionarily conserved tenet. In addition to apoptosis, other forms of cell death have been described, including necroptosis [95], pyroptosis [96], and ferroptosis [97]. Moreover, autophagic cell death is instrumental in maintaining cellular homeostasis and responding to changes in physiological conditions [98]. Four proteins, namely, histone H2A, caspase-3, caspase-7, and death effector domain-containing DNA-binding protein (DEDD), exhibit changes (either decreases or increases) in levels of monoubiquitination during apoptosis [99]. In response to diverse apoptotic stimuli, monoubiquitinated histone H2A undergoes deubiquitination [100]; however, the exact consequence of H2A deubiquitination during apoptosis remains unclear. DEDD is localized in the cytosol, nucleoplasm, and nucleolus in non-ubiquitinated, monoubiquitinated, and diubiquitinated forms [101]. Apoptosis causes a preference for monoubiquitinated and diubiquitinated forms of DEDD, whereas non-ubiquitinated DEDD diminishes. In response to proapoptotic stimuli, DEDD and caspase-3 converge on intermediate filaments, and the formation of the ternary complex triggers activation of caspase-3 and cleavage of keratin 18, resulting in the collapse of intermediate filaments [102] (Figure 3d). The level of the active histone mark H2Bub1 is downregulated in cancer, and loss of H2Bub1 promotes cancer metastasis [103]. Interestingly, p53 reduces the level of H2Bub1 by promoting the nuclear translocation of the deubiquitinase USP7, which deubiquitinates H2Bub1, leading to downregulation of its target SLC7A11 and sensitization of cells to elastin-induced ferroptotic cell death [27].

### 3.5. DNA Damage Response (DDR)

To maintain genome integrity that is essential for homeostasis, cells have evolved a set of complex mechanisms, referred to as DNA damage response (DDR) [104]. The most harmful DNA lesions are double-strand breaks (DSBs), which cause genomic instability and cancer. Phosphorylation of Ser139 of histone H2AX, named γH2AX, is a marker DSBs [105,106,107]. The E3 ligases RNF8 and RNF168 mediate monoubiquitination and K63-linked polyubiquitination of H2A/H2AX at DNA damage sites, and H2A/H2AX ubiquitination triggers the accumulation of DNA repair proteins including BRCA1 and 53BP1 [108]. Another example is that monoubiquitination of Fanconi anemia proteins FANCD2 and FANCI, mediated by the ubiquitin-conjugating enzyme UBE2T and the ubiquitin ligase FANCL, is a key step in the Fanconi anemia pathway in response to DNA damage or replication stress. Monoubiquitinated FANCD2 locks the FANCD2–FANCI complex to DNA, creating a clamp that encloses double-strand DNA and mediates the recruitment of other DNA repair proteins [109].

DNA damage tolerance promotes damage bypass to continue replication in the presence of DNA damage via two mechanisms, error-prone translesion synthesis (TLS) and error-free template switching (TS) [110]. Proliferating cell nuclear antigen (PCNA) tethers polymerases to DNA and provides a loading platform for replication factors [111]. Of note, PCNA plays a critical role in the choice of the DNA damage tolerance pathway depending on its PTMs; while monoubiquitinated PCNA results in TLS, polyubiquitinated PCNA leads to TS. Mechanistically, HLTF polyubiquitinates PCNA and RAD6–RAD18 mediates PCNA monoubiquitination. When HLTF is present, PCNA monoubiquitination is followed by polyubiquitination, resulting in TS; when HLTF is absent, monoubiquitinated PCNA triggers TLS [112] (Figure 3e). To keep error-prone TLS under control, the deubiquitinase USP1 removes monoubiquitin from PCNA. However, ultraviolet irradiation inactivates USP1 via autocleavage, leading to the accumulation of monoubiquitinated PCNA and activation of TLS [113].

Monoubiquitination also regulates cell-cycle checkpoints. p50 is monoubiquitinated in the S phase by the BARD1–BRCA1 complex. Loss-of-function mutations of the BARD1–BRCA1 complex cause deubiquitination and destabilization of p50, leading to accelerated S-phase progression and chromosomal breakage [114].

## 4. Diseases Associated with Dysregulated Monoubiquitination

Alterations of ubiquitination, including monoubiquitination, have been linked to human diseases. Among disorders associated with dysregulation of monoubiquitin signaling, five of them (Fanconi anemia, Parkinson’s disease, Charcot–Marie–Tooth disease, Noonan syndrome, and autoimmune disorder associated with facial dysmorphism) are known to carry germline mutations of E3 ligases that promote monoubiquitination. In this section, we review the two best-known genetic disorders associated with genes regulating monoubiquitination, Fanconi anemia and Parkinson’s disease.

### 4.1. Fanconi Anemia

**Figure 4:**
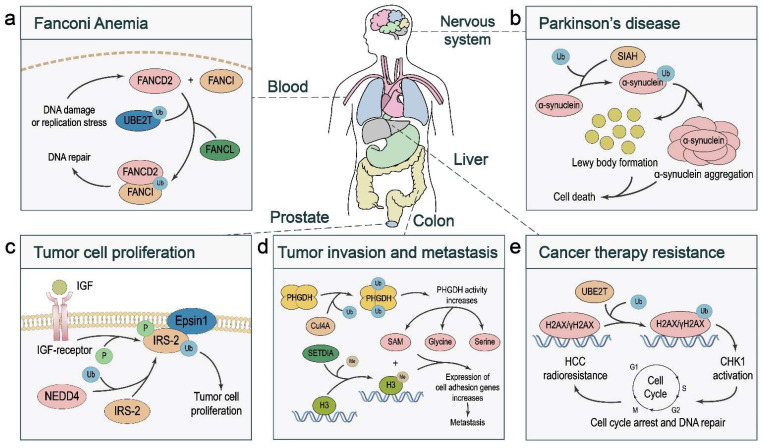
Diseases associated with dysregulated monoubiquitination. (**a**) In response to DNA damage or replication stress, the ubiquitin-conjugating enzyme UBE2T and the E3 ligase FANCL monoubiquitinate FANCD2 and FANCI, which facilitates the formation of the FANCD2–FANCI complex and DNA damage repair. In Fanconi anemia patients, impaired monoubiquitination of FANCD2 and FANCI results in DNA repair defects. (**b**) The E3 ligase SIAH monoubiquitinates α-synuclein, which promotes toxic α-synuclein aggregation, a hallmark of Parkinson’s disease. (**c**) In prostate cancer, NEDD4-induced monoubiquitination of IRS-2 enhances its association with Epsin1, which in turn promotes IGR receptor-mediated IRS-2 phosphorylation and cancer cell proliferation. (**d**) In colorectal cancer, the CUL4A-based E3 ligase complex monoubiquitinates and activates PHGDH, which increases the levels of serine, glycine, and *S*-adenosylmethionine (SAM). SAM promotes metastasis by upregulating cell adhesion gene expression via SETD1A-mediated histone methylation. (**e**) In liver cancer, UBE2T-mediated H2AX/γH2AX monoubiquitination activates CHK1 to promote DNA repair and radioresistance.

Fanconi anemia (FA) is a rare genetic disease that leads to bone marrow failure, decreased production of all types of blood cells, developmental abnormalities, and increased cancer risk. It is caused by mutations in one of the 22 Fanconi anemia complementation (FANC) genes [115]. The FA pathway is characterized by the coordinated action of the proteins expressed by these FA genes that participate in removing DNA crosslinks and other chromosomal lesions [116]. In response to DNA damage or replication stress, the ubiquitin-conjugating enzyme UBE2T and the ubiquitin ligase FANCL monoubiquitinate the FA proteins FANCD2 and FANCI, which in turn bind to DNA lesions and recruit nucleases to remove DNA crosslinks [116,117] (Figure 4a). The monoubiquitinated FANCD2–FANCI complex assumes a closed shape and creates a channel that encloses double-stranded DNA, and the ubiquitin moiety functions as a molecular pin to lock the complex on DNA [109,117]. When DNA repair mediated by the FA pathway is completed, the deubiquitinase USP1 reverses the monoubiquitination of FANCD2 [118]. In FA patients, aberrations in the FA pathway abrogate the monoubiquitination of FANCD2 and FANCI, resulting in impaired DNA repair [115].

### 4.2. Parkinson’s Disease

The loss of dopaminergic neurons in the substantia nigra and the accumulation of inclusions known as Lewy bodies are the hallmarks of Parkinson’s disease (PD). α-Synuclein plays a key role in PD pathogenesis. Dopaminergic neuron death and Lewy body formation are regarded as the result of mutations and aggregation of α-synuclein protein [119]. The ubiquitin ligase seven in absentia homolog (SIAH) monoubiquitinates α-synuclein, which promotes cytotoxic α-synuclein aggregation and Lewy body formation [120] (Figure 4b). C-terminal HSP70-interacting protein (CHIP), which has E3 ligase activity, can also monoubiquitinate α-synuclein; however, unlike SIAH, CHIP appears to selectively ubiquitinate oligomeric α-synuclein species, leading to their degradation [121]. These findings demonstrate that SIAH-mediated monoubiquitination and CHIP-mediated monoubiquitination have opposing effects on α-synuclein.

Parkin, an RBR-type ubiquitin ligase, is the second most frequently mutated protein in PD [122]. Parkin maintains mitochondrial homeostasis by mediating autophagy of damaged mitochondria (termed mitophagy) downstream of PTEN-induced kinase 1 (PINK1) [123,124,125,126,127,128]. Voltage-dependent anion channel 1 (VDAC1), which is anchored in the outer membrane of mitochondria, transports small molecules such as metabolites and calcium [129]. By promoting two different types of ubiquitination of VDAC1, the PINK1–Parkin pathway regulates mitophagy and apoptosis; while Parkin-mediated mitophagy is regulated by polyubiquitinated VDAC1, mitochondrial calcium uptake is regulated by monoubiquitinated VDAC1, which protects cells from apoptosis [130]. Notably, a PD patient-associated Parkin mutant, which cannot monoubiquitinate VDAC1, fails to rescue the PD-related phenotypes in Parkin-null flies, suggesting that Parkin-mediated monoubiquitination of VDAC1 may play an important role in PD pathogenesis [130].

## 5. Roles of Monoubiquitination in Cancer

### 5.1. Tumor Cell Proliferation and Death

Monoubiquitination can regulate cancer cell proliferation by altering the stability of oncoproteins. Estrogen receptor (ER) plays a critical role in the initiation and progression of mammary tumors, and the ER-positive subtype accounts for more than 70% of all breast cancers. The activity of ER is controlled by estrogen. Overexpression of ER leads to upregulation of oncogenic proteins including cyclin D1 and c-Myc, which stimulate breast cancer development by promoting the G1–S transition [131]. The ubiquitin ligases RNF8 [132,133] and RNF31 [134] have been shown to induce ER monoubiquitination and increase ER stability, probably by inhibiting ER polyubiquitination, leading to increased proliferation of ER-positive breast cancer cells.

Monoubiquitination can also regulate tumor growth by affecting the activity of the substrate. The binding of IGF to its receptor stimulates the tyrosine kinase activity of the IGF receptor, leading to phosphorylation of its substrates, including insulin receptor substrate (IRS)-1 and IRS-2. The hyperactivation of IRS-2 promotes the proliferation of prostate cancer cells, which is associated with NEDD4-induced monoubiquitination. Mechanistically, monoubiquitination of IRS-2 enhances its interaction with Epsin1, which in turn promotes IGF receptor-mediated IRS-2 phosphorylation [20] (Figure 4c).

Dysregulation of monoubiquitination has been linked to cancer cell death. *BAP1*, a tumor suppressor gene with frequent loss-of-function mutations or deletions in various cancer types, encodes a deubiquitinase [3]. A recent study revealed that BAP1 removes monoubiquitin from histone H2A, which in turn reduces H2A occupancy on the *SLC7A11* promoter and represses SLC7A11 expression, leading to elevated lipid peroxidation and ferroptotic cell death [25]. Consequently, loss of BAP1 in renal cancer cells results in SLC7A11 derepression, ferroptosis resistance, and tumor development [25].

### 5.2. Tumor Cell Migration, Invasion, and Metastasis

A growing body of evidence suggests that abnormalities in ubiquitination are involved in tumor invasion and metastasis [135]. Serine metabolism plays an important role in tumor progression [11]. Phosphoglycerate dehydrogenase (PHGDH) is the key enzyme in serine biosynthesis [136]. In colorectal cancer, the activity of PHGDH is enhanced by monoubiquitination at Lys146 mediated by the CUL4A-based E3 ligase complex, which increases the levels of serine, glycine, and *S*-adenosylmethionine (SAM) to promote metastasis [11]. Mechanistically, monoubiquitinated PHGDH recruits a chaperone protein to form a tetramer, which enhances the activity of PHGDH to boost serine synthesis, and the resulting overproduction of SAM upregulates cell adhesion gene expression via SETD1A-mediated histone methylation [11] (Figure 4d).

Epithelial–mesenchymal transition (EMT), a process that endows epithelial cancer cells with motility and invasiveness, is regulated by a set of EMT-inducing transcription factors downstream of microenvironmental stimuli such as TGF-β [137]. Epithelial cells are tightly united by cell–cell junctions, especially tight junctions (TJs) and adherens junctions (AJs); the latter is mediated by the transmembrane protein E-cadherin and cytoplasmic catenin proteins including α-catenin and p120-catenin. In response to stimulation by TGF-β, the E3 ligase SMURF1 monoubiquitinates phosphorylated p120-catenin, leading to the dissociation of AJs, EMT, and breast cancer metastasis [138].

Notably, monoubiquitination of the core components of the TGF-β pathway is instrumental in tumor cell migration and metastasis. As mentioned above, monoubiquitination of SMAD4 blocks the interaction of SMAD4 with phospho-SMAD2 (Figure 3c), and the deubiquitinase USP9X reverses this PTM to activate TGF-β signaling, which in turn promotes migration and metastasis of breast cancer cells [84,86]. Moreover, monoubiquitination of R-SMADs inhibits their DNA-binding ability, and the deubiquitinase USP15 facilitates TGF-β-induced breast cancer cell migration by antagonizing R-SMAD monoubiquitination [139].

Certain tumor subtypes are more aggressive than other subtypes of the same cancer. For example, thyroid cancer is classified into three subtypes: well-differentiated thyroid cancer (WDTC), poorly differentiated thyroid cancer (PDTC), and undifferentiated/anaplastic thyroid cancer (ATC). ATC is one of the most aggressive solid tumors, representing 2–5% of thyroid cancer [140]. TRIM11, an E3 ligase that belongs to the tripartite motif (TRIM) protein family, has been linked to the malignant phenotypes of ATC [141]. Mechanistic studies have indicated that TRIM11 can substantially increase the monoubiquitination of YAP, the key downstream effector in the Hippo pathway, while inhibiting K11- and K48-linked polyubiquitination of YAP, leading to elevated ATC cell migration [141].

### 5.3. Cancer Therapy Response

Aberrant PTMs are involved in cancer cell resistance to chemotherapy, radiotherapy, and immunotherapy. The effectiveness of radiotherapy depends on the induction of DNA double-strand breaks (DSBs), while tumor cells can upregulate DDR, which enhances DSB repair to confer resistance to radiotherapy [142]. In liver cancer cells, UBE2T-mediated H2AX/γH2AX monoubiquitination activates cell-cycle checkpoint kinase 1 (CHK1) to induce cell-cycle arrest, which allows effective DNA repair, leading to radioresistance [143] (Figure 4e).

Ovarian cancer often exhibits hypersensitivity to the chemotherapeutic agent cisplatin, which could be caused by disruption of the Fanconi anemia (FA)–BRCA pathway [144]. Monoubiquitination of FANCD2 is a measure of the activity of this DNA damage repair pathway, and disruption of this pathway in ovarian cancer results from the hypermethylation and silencing of one of the FA genes, *FANCF*, leading to increased sensitivity to the DNA-damaging agent cisplatin [144]. Ovarian cancer cells can restore this pathway through demethylation and derepression of *FANCF*, resulting in acquired cisplatin resistance [144].

Natural killer T (NKT) cells are a special subset of T lymphocytes [145]. The interaction of the NKT cell receptor with glycolipid antigens such as α-galactosylceramide (α-GalCer) presented by the MHC class I-like protein CD1d activates NKT cells, resulting in the fast production of cytokines such as IFNγ and IL-4 [146]. On the other hand, however, repeated injection of α-GalCer causes long-term unresponsiveness or anergy [147]. In NKT cells, the E3 ligase Cbl-b promotes monoubiquitination of CARMA1, a signaling molecule in the NF-κB pathway, resulting in failed tumor rejection. On the basis of this work, combination treatment with Cbl-b inhibitors and α-GalCer may improve the clinical application of NKT cells [147].

## 6. Conclusions

The functions of monoubiquitination in homeostasis and cancer are still understudied. Compared with the well-established role of polyubiquitination in proteasomal degradation, monoubiquitination acts as a more versatile signal and could have functional roles in diverse biological processes. This PTM can either prevent or promote proteolytic polyubiquitination. Furthermore, monoubiquitinated proteins may serve as signaling platforms. By modulating the activity, subcellular localization, protein–protein interactions, or endocytosis of the substrate, monoubiquitination regulates gene transcription, endocytosis, signal transduction, cell death, and DNA damage response, thereby playing important roles in cellular and organismal homeostasis. Dysregulation of monoubiquitination contributes to the pathogenesis of neurodegeneration and cancer. There is a growing interest in targeting specific E3 ligases or DUBs for therapy. A better understanding of the regulations and regulators of monoubiquitination would inform the development of inhibitors of E3 ligases or DUBs.

## Data Availability

Not applicable.
